# Predicting Adverse Health Outcomes in Long-Term Survivors of a Childhood Cancer

**DOI:** 10.3390/children1020063

**Published:** 2014-07-15

**Authors:** Chaya S. Moskowitz, Kevin C. Oeffinger

**Affiliations:** 1 Department of Epidemiology and Biostatistics, Memorial Sloan Kettering Cancer Center, 307 E 63rd Street, New York, NY 10065, USA; 2 Departments of Medicine and Pediatrics, Memorial Sloan Kettering Cancer Center, 300 E 66th Street, 9th Floor, New York, NY 10065, USA; E-Mail: oeffingk@mskcc.org

**Keywords:** clinical prediction tool, risk prediction model, late effects, second cancers

## Abstract

More than 80% of children and young adults diagnosed with invasive cancer will survive five or more years beyond their cancer diagnosis. This population has an increased risk for serious illness- and treatment-related morbidity and premature mortality. A number of these adverse health outcomes, such as cardiovascular disease and some second primary neoplasms, either have modifiable risk factors or can be successfully treated if detected early. Absolute risk models that project a personalized risk of developing a health outcome can be useful in patient counseling, in designing intervention studies, in forming prevention strategies, and in deciding upon surveillance programs. Here, we review existing absolute risk prediction models that are directly applicable to survivors of a childhood cancer, discuss the concepts and interpretation of absolute risk models, and examine ways in which these models can be used applied in clinical practice and public health.

## 1. Introduction

There are over 379,000 people in the United States who are living with or beyond a childhood or adolescent cancer diagnosis [1]. With improvements in treatment over the last decade, five-year survival rates now exceed 80% leading to an increasing number of long-term survivors of a pediatric cancer [2]. These long-term survivors may experience multiple health problems as a result of the treatment they received. Previous studies have demonstrated increased risks of second cancers, heart disease, pulmonary problems, cognitive dysfunction, and other medical conditions [3,4,5,6,7]. Many of these conditions are associated with morbidity and premature mortality. However, for a number of these adverse health outcomes, the associated morbidity and mortality can be reduced or possibly even prevented by early detection using existing screening and surveillance methods or though preventive strategies. 

This observation has led to a call to provide childhood cancer survivors with risk-based care where the precise nature and components of the follow-up care are directed by an individual’s risk of developing a specific health condition [8,9,10,11]. Consequently, identifying individuals at high risk of an outcome for which there is an effective method of early detection and intervention or an effective prevention is critical [12]. To a large extent, these risks are driven by cancer therapy but aspects such as genetic predispositions and other personal factors may play a role. An understanding of how these different elements jointly contribute to the risk of a health problem can therefore be used to guide follow-up care.

Clinical prediction models or rules that are based on combinations of risk factors are one important and frequently used tool for identifying individuals at highest risk of a health problem. These models are typically developed with the goal of providing individualized estimates of the condition occurring and are constructed specifically so as to be useful in clinical practice. There are some prominent examples of prediction models informing clinical practice and health care policy in the general population. For instance, the Framingham risk score and a newly created American College of Cardiology/American Heart Association risk calculator both predict the risk of future cardiovascular disease and have been used to guide interventions including the use of statin therapy [13,14]. The Gail model and Claus model predict the risk of developing breast cancer and have been used to facilitate patient communication, to guide clinical decisions, and to design intervention studies [15,16]. 

Here, we review clinical prediction models that were developed specifically for use in survivors of a childhood cancer. We provide a brief synopsis of the concept of clinical prediction models and discuss opportunities for future work in this area. 

## 2. Constructing Clinical Prediction Models

**Figure 1:**
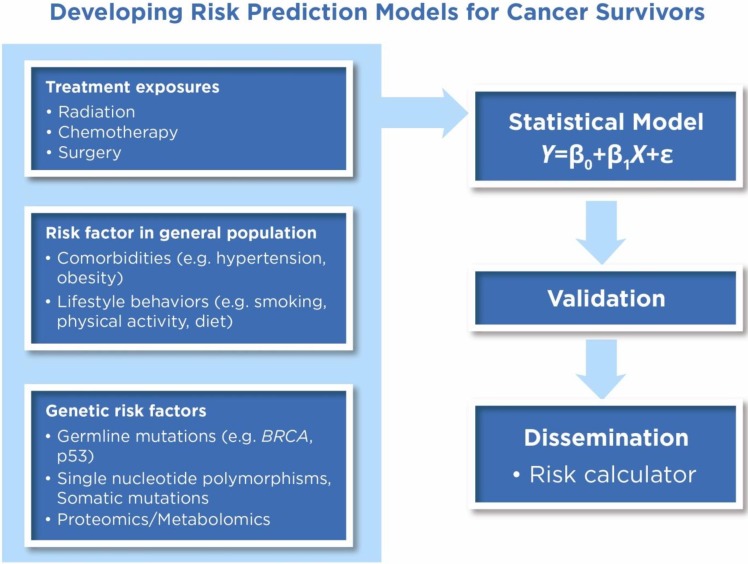
Developing risk prediction models for cancer survivors.

Over the last decade or so, risk prediction modeling has received much attention, and many different models have been constructed to predict a wide variety of outcomes. The methodology to develop a prediction model and implement it in practice is generally applicable regardless of the medical setting or outcome of interest. An overview of the process is presented in Figure 1. 

There is much existing literature on this topic, and there is ongoing work on developing new methods for constructing and validating prediction models. We do not provide a comprehensive review of the methodology or extensive literature, but refer the interested read to several texts on this topic [17,18,19,20,21]. Instead, we discuss some basic concepts and issues that warrant careful consideration when building and applying prediction models to childhood cancer survivors. 

### 2.1. Model Development

Clinical prediction models are usually based on multivariable regression models (mathematical functions combining risk factors) that provide the absolute risk of developing a condition within a given time frame. Absolute risk, also referred to as the crude or cumulative or individualized risk, is the probability that a person with a given set of characteristics will develop and be diagnosed with a health condition in a specified period of time. Time is usually measured using age as the scale, but for childhood cancer survivors it may also be relevant to use the interval since childhood cancer diagnosis as the time scale. For instance, we might look at individuals who are currently 40 years old, do not have the health condition, and predict the risk of it developing by age 50, or we might look instead at individuals who are alive and free of the condition 15 years after their childhood cancer diagnosis and predict the risk of it occurring in the next 10 years. 

Prediction models are formed by combining factors (also called predictors) that are associated with the outcome in an equation. A model’s ability to accurately project an outcome depends critically on the predictors included in the equation and their ability to capture the major contributors to an individual’s risk. As a result models developed for use in people who do not have a history of childhood cancer are usually not applicable to childhood cancer survivors who tend to have very different risk profiles due to the unique exposures from their cancer therapy. 

Prediction models constructed for use in childhood cancer survivors can include three broad classes of risk factors: treatment-related exposures, traditional risk factors identified in the general population, and genetic risk factors. While one might be inclined to focus exclusively on the treatment-related exposures because they do tend to modify the chance of an adverse health outcome to a very large degree, it would be remiss to not consider the potential of traditional risk factors in people without a prior childhood cancer to also modify risk in childhood cancer survivors. We highlight some specific examples of these types of risk factors in Figure 1. In deciding how to quantify predictors and define the variables representing the risk factors to be included in a model it is important to only use information that would be available in clinical practice. For example, radiation dosimetry should not be used as a measure of radiotherapy in a model because it is generally not available in patients’ medical records; instead, the information that is typically included in a treatment summary, such as prescribed doses and radiation fields, would be appropriate.

### 2.2. Interpretation of Model Estimates

Despite the fact that estimates from clinical prediction models are at times referred to as “individualized” estimates, it is important to keep in mind that the numbers obtained from such models are not estimating the chance that a particular person will experience the outcome. For example, if a patient’s characteristics are input into a model predicting cardiovascular disease in the next 10 years and it yields an estimate of 11%, we should not interpret this to say the patient has an 11% chance of developing cardiovascular disease within 10 years. Rather, the correct way to interpret the estimate is to consider 100 people with the same risk factors as this particular patient. Out of the 100 such people, 11 of them will develop cardiovascular disease in the next 10 years. Moreover, at least within the clinical and research communities, one should keep in mind that these are estimates that can vary. That is, if a different group of 100 people with the same risk factors was chosen, for instance, it may be that instead 10 or 12 of them ultimately develop cardiovascular disease. This uncertainty or variation is reflected in confidence intervals that are usually presented in publications. Recent publications have highlighted the difficulty in trying to communicate uncertainty to patients, pointing to the inherent difficulty in this task [22,23]. 

### 2.3. Model Validation

Once the model has been developed, it is imperative to evaluate its performance and validate it before using it in any way. While there is on-going discussion in the statistical literature on the best metrics to use for quantifying predictive ability, everyone agrees on the importance of validating the model and, in particular, validating it in external data. Validation involves taking the prediction model, applying it to a group of people to obtain estimates of their risk of the event of interest, and then comparing these estimates with their actual outcomes to see how well the model is able to predict the events. There are two components to assessing a model’s predictive accuracy: discrimination and calibration. Discrimination refers to how well the model distinguishes between people who develop the outcome being predicted and those who do not. Calibration measures how well the predicted probabilities of the outcome match the observed events. 

Evaluating how well the model works using data on the same people used to develop it can result in concluding that the model has better predictive ability than it truly does; hence, the need to test it out on new people. In childhood cancer survivors, this may be particularly challenging given that childhood cancer is relatively rare and obtaining data on a sufficient number of people to both develop a model and then externally validate it in a meaningful way can be difficult. There is both the potential and the need for collaboration and sharing of data across countries and studies to produce useful prediction models applicable to this population. 

### 2.4. Model Dissemination

A final important step to consider is how the resulting model will be shared with the clinical, research, and patient communities. What form will the final product take that will facilitate use by its intended audience? For publications one could present an equation, tables showing estimates for patients with certain risk profiles, a graphical representation of the model (nomograms being one of the more popular of these [19,24]), or a combination of these. In addition, though, online, interactive tools, sometimes referred to as risk calculators, are becoming increasingly more common and can be instrumental in providing access and easy use of the prediction tool by both clinicians and patients. (For some examples, the National Cancer Institute maintains a website with examples of existing online prediction tools [25]. Precisely how to communicate information on individualized risk estimates so that they are easily understandable by patients warrants careful consideration when developing an online tool. Fagerlin and colleagues review some possibilities and provide suggestions on how best the communicate information on health risks [22].

## 3. Existing Clinical Prediction Models for Childhood Cancer Survivors

Despite the many existing cancer risk prediction models, there are surprisingly few clinical predictions models that are applicable to survivors of a childhood cancer. To a large extent, this is likely a reflection of when evidence regarding the multiple health conditions faced by childhood cancer survivors started to become apparent. Before prediction tools are developed, there needs to be awareness that a population faces a specific health problem and epidemiologic studies that identify risk factors need to be conducted. Only over the last decade or so has the state of research into the health conditions faced by childhood cancer survivors reached the point where clinical prediction models that translate solid scientific evidence into public health practice are a natural next step.

There are only two models of which we are aware that are developed specifically for childhood cancer survivors, and they both predict second primary neoplasms. The first model, by Travis and colleagues, predicts the risk of breast cancer in women who are survivors of Hodgkin lymphoma [26]. The authors studied Hodgkin lymphoma (HL) survivors who were treated for HL before age 30, between 1965 and 1994, and had survived at least one year. Using data from a population-based nested case-control study that included 105 HL survivors with breast cancer and 266 controls without breast cancer, the model includes information on the total radiation dose to the mediastinum, alkylating agent use, age at Hodgkin lymphoma diagnosis, and current age, as well as information on death from causes other than breast cancer. The model predicts the absolute risk of breast cancer up to 30 years after the Hodgkin lymphoma diagnosis and shows that breast cancer risk increases with current age, time since HL diagnosis, and radiation dose. The predictions from the model are primarily conveyed through the use of tables in the manuscript. Notably, the authors used only information that would be found in medical records but did not have information available in their data on breast cancer risk factors in the general population such as family history of breast cancer or reproductive factors.

The second model (or rather, set of models as the authors present three different models) predicts thyroid cancer in survivors of a childhood cancer. Combining data on 12,150 five-year survivors of a childhood cancer who were either participants in a large North American cohort study, a North American nested case-control study, and a Nordic nested-case control study, Kovalchik *et al.* utilize information on radiotherapy use, alkylating agent use, childhood cancer diagnosis, birth year, age at diagnosis, gender, and past thyroid nodule diagnosis to predict the absolute risk of a future second primary thyroid cancer [27]. With 159 cases of thyroid cancer, they show results for three different models which differ by the level of information that may be available for use in the model. Their first model includes information that would be available from patients’ self-reporting only, the second model adds in predictors obtainable from medical records, and the third model incorporates reconstructed radiation doses which are generally not available in clinical practice. They found that the second model performed substantially better that the first model and just as well as the third model which included a measure of radiation exposure that is considered more accurate than information available from medical records. They validate all three models on an external French cohort of 3,254 five-year survivors of childhood cancer and assess model performance by evaluating the models’ discrimination and calibration. 

## 4. Directions of Future Work

Given the very limited number of clinical prediction tools directly applicable to childhood cancer survivors and the considerable morbidity and mortality that childhood cancer survivors face from long-term effects of their cancer treatment, this is an area that is ripe for future research. Conditions for which risk prediction models may be useful include any long-term health outcome or late-effect for which there is an effective method for testing for the condition and early detection or available treatment improves patients’ outcomes [12]. Broadly, these conditions could include second malignant neoplasms, cardiac toxicities, and endocrine/metabolic disorders. In Table 1, we list some examples of specific late effects for which clinical prediction tools could be useful. Current work that is underway includes risk prediction of breast cancer in females treated with chest radiation and risk prediction of heart failure post cardiotoxic therapy.

For each of these outcomes there are some established risk factors that can be considered for inclusion in prediction models. These include chest radiotherapy details for breast cancer and cardiovascular disease, cranial radiotherapy details for meningioma, stroke, and endocrinopathies, abdominopelvic radiotherapy information for colorectal and gastric cancer, and anthracycline use for cardiovascular disease. There are also other risk factors that should be evaluated for inclusion in a model, though, factors which may currently be unknown and others which are not well-established or for which there is conflicting evidence. For instance, we have included genetic risk factors in Figure 1. While many experts agree that genetic factors are likely to play an important role in late effects, this is an emerging field for which we still know very little. As information becomes available, it can be considered for inclusion in a prediction model. Other examples of factors where their contributions to the risk of developing late effects are less certain include examples include oral contraceptive/hormone use for breast cancer, anthracycline use for insulin resistance, diet and body mass index for virtually all of the outcomes listed in the table. 

**Table 1:** Outcomes for potential prediction tools for childhood cancer survivors.

Second neoplasms
● Breast cancer
● Colorectal cancer
● Gastric cancer
● Meningioma
**Cardiovascular disease**
● Coronary artery disease/Myocardial infarction
● Cardiomyopathy/Heart failure
● Valvular heart disease
● Stroke
**Endocrinopathies**
● Insulin resistance/diabetes
● Osteoporosis
● Lipid disorders
● Metabolic syndrome

## 5. Conclusions

Approximately three quarters of adult survivors of a childhood cancer treated in the 1970s, 1980s, or 1990s will develop a chronic condition by age 40 and close to half will experience a serious health condition [5,28]. Although the incidence of late effects for survivors treated more recently is not expected to be quite as high, improvements in treatment combined with a slow increase in the incidence of childhood cancer continue to contribute to an increasing number of childhood cancer survivors who may still be at risk for various sequelae related to their cancer or therapy [29]. Identifying at-risk groups within the childhood cancer survivor population is an important component of providing survivorship care, particularly since not all survivors require intensive follow-up while others may substantially benefit from such care. Appropriately constructed clinical prediction models can facilitate incorporating scientific evidence into the delivery of survivorship care at several different levels. 

At the population level, prediction models can be instrumental for informing guidelines grounded in empirical evidence. There have been multiple calls for basing clinical practice guidelines for cancer survivors on risk stratification tools that can identify those survivors at increased risk of specific late effects [9,10,12,30]. At the level of the healthcare provider, prediction models should not obviate but could rather supplement clinicians’ reasoning and help guide decision making about appropriate care and management [31]. They can also be employed to counsel patients by informing them of their risks of developing a condition. At the patient level, they may help patients make complex medical decisions and can facilitate shared patient-physician decision making [22,31]. Finally, prediction models can also be useful in research by identifying high-risk patients for enrollment into clinical trials and potentially decreasing the number of participants that need to be enrolled in order to study the efficacy of a new therapy. 

The study of risk factors for late effects is an evolving field. Just as there are almost certainly unidentified factors that modify the risk of adverse health outcomes in survivors who were treated with regimens no longer in use, there is still much to be learned about factors that modify the risk of these same, as well as other, health outcomes in childhood cancer survivors who were treated with contemporary regimens. As the science progresses, existing risk prediction models can be updated to incorporate new information [32,33]. Indeed, this is an important consideration if clinical prediction tools are to maintain their utility. 

In conclusion, there are multiple long-term health outcomes related to childhood cancer and its therapy which adversely affect childhood cancer survivors. Many of these outcomes are modifiable either through early detection by screening coupled with effective treatment or through preventive strategies such as changing lifestyle behaviors or chemoprevention. In contrast to the number of late effects facing childhood cancer survivors there are relatively few clinical prediction tools directly applicable to this population. Using existing methods together with some careful thought given to the information input to and output from the model, newly clinical prediction tools have the potential to address an unmet need in cancer survivorship research and delivery of care. 
